# The Role of Age in the Abdominal Aortic Aneurysm Repair

**DOI:** 10.5935/1678-9741.20160044

**Published:** 2016

**Authors:** Henrique Murad, Felipe Francescutti Murad

**Affiliations:** 1 Universidade Federal do Rio de Janeiro, Rio de Janeiro, RJ, Brazil.; 2 Serviço de Cirurgia Vascular do Hospital Federal de Ipanema do Ministério da Saúde, Rio de Janeiro, RJ, Brazil.

Age is a factor to be considered in any minimally invasive new surgical procedure like endovascular aneurysm repair (EVAR). There is a growing policy of using less invasive procedures looking for rapid recovery, less blood transfusion, less surgical trauma, less morbidity and less mortality. The two major questions that need to be answered when we are faced with endovascular treatment of aneurysms (AAA) are: 1. Can we offer surgical treatment to older patients, otherwise not candidates for surgery, with low surgical mortality and morbidity?; 2. Are the results equivalent to those obtained with the open procedure, so we can offer EVAR even to younger patients?

Those questions are issued in the paper "Is age a determinant factor in EVAR as a predictor of outcomes or in the selection procedure? Our experience"^[1]^. The authors have conducted a retrospective study of 171 patients submitted to EVAR for the treatment of AAA divided in three groups: under 70 years of age (55 patients), between 70 and 80 (65 patients) and over 80 years of age (51 patients). The surgical mortality was 1.2%. They had no 30-day deaths in the groups under 70 and between 70 and 80 years of age, but there were 2 deaths in the group over 80 years of age. They have found no statistically difference on surgical mortality, incidence and type of endoleak, aneurysm sac behavior or reinterventions among the groups.

The majority of patients had high surgical risk (ASA was III and IV in 84.6% of patients); in the group of the patients over 80 years of age, the surgical mortality was 3.9% and the median survival in this age group was 8.3 years. They have clearly demonstrated that old age per se is not a contraindication for the EVAR treatment of AAA.

Taking into account the younger patients, there are some considerations to be done. Even though there is at least one 38 years old patient, we don't know the median age of the 55 patients belonging to this group. Some patients were followed up for twelve years, but this time period could be too short to evaluate the long-term result in a patient in their forties. The mortality and reintervention rate were not different between the groups. Reintervention could be a problem due to the risk of death, but that was not the case in this group of patients. In Altaf et al.^[2]^ study, with 165 patients less than 65 years of age submitted to AAA repair, being 97 EVAR and 68 open surgeries, they found almost 40% late mortality in 6 years, surgical procedure mostly from non-aneurismal causes. They suggest that young patients with AAA have a shorter life expectancy than the age-matched population without AAA. Probably AAA in younger age is a marker for more aggressive vascular disease.

There is still a question to be answered: What is the procedure to be used in low risk patients with AAA under 50 years of age?

Patients under 70 years of age had less erectile dysfunction than the other groups, but no conclusion could be drawn from it, because the authors didn't performed any study addressed to this kind of dysfunction in the postoperative period. Koo et al.^[3]^ studying a small number of patients (26 open repair and 21 EVAR) between 1 and 2 years after AAA repair, found greater increase in the sexual dysfunction in the open group than in the EVAR group, but the numbers are small and all the patients started with high preoperative sexual dysfunction. Probably this is due to the fact that EVAR does not interfere with the parasympathetic nervous system.
